# NURSING HOME ADMINISTRATOR EXPERIENCES DURING COVID-19: AN EXPLORATORY STUDY

**DOI:** 10.1093/geroni/igac059.1783

**Published:** 2022-12-20

**Authors:** Susanny Beltran, Jennifer Wagner, Vivian Miller

**Affiliations:** University of Central Florida, Orlando, Florida, United States; Bowling Green State University, Bowling Green, Ohio, United States; Bowling Green State University, Bowling Green, Ohio, United States

## Abstract

Nursing homes have been the epicenter for the COVID-19 pandemic; 149,107 residents and over 2,200 staff have died of COVID-19. In addition to the loss of lives, 99% of nursing homes report staffing shortages. Various exploratory studies have emerged gathering the experiences of frontline nursing home staff during COVID-19, however less is known about the experiences of Nursing Home Administrators (NHA) responsible for overseeing personnel and operating a facility in line with shifting state and federal mandates. Thus, this study explores the experiences of NHA during the pandemic. A cross-sectional online survey was conducted. In addition to demographic and facility-level questions, open-ended questions explored prior training on infection prevention, day-to-day operational challenges, needs, and considerations of leaving their role as administrator. The total sample (N=60) included 47 NHA of record and 13 assistant administrators/other; 53% worked in corporate NHs and 23% were part of continuing care retirement communities. Respondents report prior infection prevention training, but indicate it was not adequate preparation for COVID-19. Moreover, administrators describe challenges in recruiting and retaining staff, and in supporting staff mental health needs (e.g., burnout, PTSD). The majority of NHAs endorse a desire to step away from their role, but indicate a commitment to residents keeps them from resigning. Findings indicate that NHAs, like other members of the NH team, have experienced the effects of COVID-19, and point to specific training and support needs to equip NHAs for work in the context of this pandemic and future emergencies.

